# Understanding geriatric models of care for older adults living with HIV: a scoping review and qualitative analysis

**DOI:** 10.1186/s12877-023-04114-7

**Published:** 2023-07-08

**Authors:** Kristina Marie Kokorelias, Anna Grosse, Alice Zhabokritsky, Luxey Sirisegaram

**Affiliations:** 1 Division of Geriatric Medicine, Department of Medicine, Sinai Health System and University Health Network, Suite 475 - 600 University Avenue, Toronto, ON M5G 1X5 Canada; 2 Department of Occupational Science & Occupational Therapy, Temerty Faculty of Medicine, University of Toronto, 160 - 500 University Ave, Toronto, ON M5G 1V7 Canada; 3 Rehabilitation Sciences Institute, University of Toronto, Toronto, Canada; 4 Division of Geriatric Medicine, Department of Medicine, Medical Sciences Building, University of Toronto, 1 King’s College Cir, Toronto, ON M5S 1A8 Canada; 5 Department of Medicine, Medical Sciences Building, The University of Toronto, King’s College Cir, Toronto, ON M5S 1A8 Canada; 6 Infectious Diseases, Department of Medicine, University Health Network, 610 University Ave, Toronto, Toronto, ON M5G 2M9 Canada; 7 CIHR Canadian HIV Trails Network, 570-1081 Burrard Street, Vancouver, BC V6Z 1Y6 Canada

**Keywords:** Geriatrics, Models of care, Older adults, Qualitative, Scoping review

## Abstract

**Background:**

Advances in Human Immunodeficiency Virus (HIV) treatment have reduced mortality rates and consequently increased the number of individuals with HIV living into older age. Despite this, people aged 50 years and older have been left behind in recent HIV treatment and prevention campaigns, and a gold-standard model of care for this population has not yet been defined. Developing evidence-based geriatric HIV models of care can support an accessible, equitable, and sustainable HIV health care system that ensures older adults have access to care that meets their needs now and in the future.

**Methods:**

Guided by Arksey & O’Malley (2005)’s methodological framework, a scoping review was conducted to determine the key components of, identify gaps in the literature about, and provide recommendations for future research into geriatric models of care for individuals with HIV. Five databases and the grey literature were systematically searched. The titles, abstracts and full texts of the search results were screened independently in duplicate. Data were analyzed using a qualitative case study and key component analysis approach to identify necessary model components.

**Results:**

5702 studies underwent title and abstract screening, with 154 entering full-text review. 13 peer-reviewed and 0 grey literature sources were included. Most articles were from North America. We identified three primary model of care components that may improve the successful delivery of geriatric care to people living with HIV: Collaboration and Integration; Organization of Geriatric Care; and Support for Holistic Care. Most articles included some aspects of all three components.

**Conclusion:**

To provide effective geriatric care to older persons living with HIV, health services and systems are encouraged to use an evidence-based framework and should consider incorporating the distinct model of care characteristics that we have identified in the literature. However, there is limited data about models in developing countries and long-term care settings, and limited knowledge of the role of family, friends and peers in supporting the geriatric care of individuals living with HIV. Future evaluative research is encouraged to determine the impact of optimal components of geriatric models of care on patient outcomes.

**Supplementary Information:**

The online version contains supplementary material available at 10.1186/s12877-023-04114-7.

## Background

Human immunodeficiency virus (HIV) continues to be characterized as one of the most prominent public health threats [1], although advances in antiretroviral therapy (ART) have reduced mortality rates and transformed HIV into a manageable, chronic disease [2]. The life expectancy for people living with HIV who have had early and sustained access to ART is now similar to that of HIV-negative populations [3–5]. Thus, there is now an increase in the number of individuals living with HIV into older age [6] and the number of older adults (aged ≥ 50 years [7]) living with HIV is expected to increase even further in the coming years [8]. The proportion of older adults living with HIV has nearly tripled since 2000 [9].

Older adults with HIV have an increased risk of dementia, diabetes, frailty, depression, osteoporosis, and some cancers, compared to those who are HIV negative [10–12]. Comorbidities commonly associated with ageing (e.g., diabetes) have been found to increase the risk of opportunistic infections (e.g., HIV-related concerns) in older adults with HIV [13–16]. Moreover, stigma is associated with higher rates of loneliness, social isolation and depression in the HIV population [17]. Despite their increased risk of poor health and social outcomes, older adults living with HIV face many challenges accessing appropriate health and social care, further exacerbating their poor health outcomes [18]. The stigma associated with HIV may result in a fear of disclosure that delays treatment [19], and individuals with HIV can feel discriminated against by healthcare providers, resulting in hesitation about or refusal to seek medical care [20, 21]. Older adults also tend to not access social services designed for the HIV-infected population because of their own assumption that these programs are created only for younger individuals [22]. Consequently, HIV scholars have urged for a health and social care system where knowledge and communication about geriatric HIV care are encouraged amongst advocates who work directly with this population, such as geriatric healthcare workers [23].

Geriatric specialists have expertise in managing many comorbidities that share associations with both ageing and HIV, despite geriatricians being hesitant to take a prominent role in the care of HIV in older adults [24] due to a lack of experience and training [25]. While health policy reports a preference for general practice-based HIV care over specialist care [26, 27], general practitioners may have a less nuanced understanding about the holistic care of an older adult with complex comorbidities, geriatric syndromes, and metabolic complications when compared with geriatricians [28]. The use of the Comprehensive Geriatric Assessment (CGA) has been explored, and may lead to improved health and social outcomes in the older adult-HIV population [6–35], and may be used to measure outcomes in clinical trials that aim to improve the delivery of HIV care for the older adult-HIV population [36]. However, in the absence of specialized geriatric models of HIV care, many older adults with HIV fail to receive a CGA [37, 38] and the recommendations from CGAs are rarely implemented due to a lack of feasibility following a geriatric consult for older adults with HIV [39].

Numerous models of care, defined as “the way health services are delivered” [40] (pg., 3), have been developed for older adults with HIV. Many involve geriatric specialists in HIV care, with geriatricians taking on various responsibilities ranging from consultation to leadership roles [36, 41]. However, the gold-standard model of care for older adults living with HIV have not yet been defined [34, 35], and geriatric care is often delivered by non-geriatric specialists [16]. Instead of examining models of care, recent literature reviews have tended to focus on the prevalence and experiences of older adults in HIV care [7, NaN], or the experiences of geriatricians [24]. As implementing geriatric models of HIV care into healthcare settings requires unique considerations [28], an improved understanding of existing models of care may inform best-practices. This approach has been done to inform the design and delivery of other models of healthcare [42–45]. Therefore, we conducted a scoping review of the existing evidence about geriatric models of care for older adults within the context of HIV. To our knowledge, this is the first review to systematically identify the core operational components of existing models of care specific to older adults living with HIV.

## Methods

A scoping review was selected to map the available literature on geriatric models of care for older adults within the context HIV [46]. The protocol for our scoping review followed the well-established framework outlined by Arksey and O’Malley [46] and later refined by Levac et al. [47] and Colquhoun et al. [48]. The framework was selected as it provides guidance to ensure a rigorous scoping review approach utilizing a comprehensive search strategy [46]. Our protocol has been published elsewhere (blinded for review #1) but is briefly described within this section of the manuscript. There were no deviations from our protocol. The framework includes five steps: 1) identifying the research questions; 2) identifying relevant literature; 3) study selection; 4) charting the data; 5) collating, summarizing and reporting the results [46]. The optional sixth step of consulting with key stakeholders was not followed due to financial resource constraints. We briefly summarize each step and report our findings in accordance with The Preferred Reporting Items for Systematic reviews and Meta-Analyses (PRISMA) Extension for Scoping Reviews (PRISMA-Scr) [49] (see Supplemental Material A).

### Step 1: Identifying the research questions

Our questions were developed to support a knowledge synthesis that could mobilize the current evidence into practice. Our study aimed to answer: What are the key components of the existing models of HIV care for older adults (aged ≥ 50 years [7, 29])?

### Step 2: Searching for relevant studies

To identify studies, we developed a comprehensive search strategy with an experienced medical information specialist (CDC) who first conducted the search in MEDLINE(R) ALL (in Ovid, including Epub Ahead of Print, In-Process & Other Non-Indexed Citations, Ovid MEDLINE(R) Daily) and then translated it into NLM’s PubMed OVID Embase + Embase Classic, EBSCO’s CINAHL Complete, Clarivate’s Web of Science Core Collection, and Elsevier’s Scopus from the earliest record to 2022 (see Supplemental Material B for the full strategies**)**. The search strategy was peer-reviewed according to the peer-review of electronic search strategy guidelines (the PRESS strategy) [50]. MeSH terms were used. All searches were limited to English language. The final searches were completed on Friday, October 21, 2022. Duplicates were removed using the Bramer method in EndNote [51]. Covidence was used to manage the review process, including the deduplication of database results [52].

Gray literature and non-indexed articles were searched for using Google Scholar, Open Grey, open Google searches and relevant websites, including the World Health Organization, UK National Research Register, CADTH’s “Grey Matters”, New York Academy of Medicine's Grey Literature Report, the Canadian Medical Association InfoBase and the National Institute for Heath and Care Excellence – Guidance. Similar search terms used in the scientific search were used. We also consulted with stakeholders of our research (i.e. geriatricians, infectious disease specialists) for any gray literature missed.

### Step 3: Selecting studies

Three reviewers (LS, KMK and AG) independently screened article titles and abstracts (level 1-screening) and then full articles (level 2-screening) were screened in duplicate to identify potentially relevant studies. In both levels of screening, any disagreements were resolved through team-based discussion. Articles were included if they described an implemented model or models of care to treat older adults living with HIV exclusively (i.e., not as part of the treatment for multi-morbidity including HIV) and included a registered healthcare provider that specialized in geriatric care (e.g., gerontology social worker, geriatric clinical nurse specialist, geriatrician). Perspective (viewpoint) papers that describe implemented models of HIV care were also included. Book sections, theses, film broadcasts, abstracts without adequate data, and literature reviews were excluded. Articles were also excluded if they: (1) did not propose an original model of HIV care specifically for older adults (i.e., models of care for all adults or models that may include older adults), (2) focused on ethical issues or the theoretical understandings of HIV care or geriatric care, (3) focused on training healthcare providers on how to deliver HIV and/or geriatric care; and (4) described social support, rather than care in a clinical, health-care context. Forward and backward searching were conducted on the final full-text articles to ensure a broad search using EndNote and Citationchaser [53, 54].

### Step 4: Charting the data

The same three reviewers independently extracted data from the included studies using a data abstraction form that was developed and pilot tested by two researchers (LS and KMK). The data form was tested on five articles for consistency in understanding and ensuring that all relevant data was captured. No changes were made after comparing the pilot test results. The fields for abstraction included author last name, year, study type, setting, geographic location (country), methodology, characteristics of intervention (model of care) and delivery method, participant and provider characteristics, patient inclusion and exclusion criteria, desired outcomes (primary and secondary), results and key conclusions.

### Step 5: Collating, summarizing and reporting the results

Data were analyzed using a systematic qualitative case study analytic approach [55]. First, each author reviewed the abstracted data and independently noted the core operational components (i.e., model structure and process for delivery) described in the models of care. Then the authors came together to list all the identified model components across the included articles, by exploring the similar and different terms to describe the same model components. Each model component was given a label and a definition. These components became the basis of codes that were then appropriately applied by one author (KMK) to each article using NVivo 12 software [56]. Next the coded data was reviewed by all authors to determine how each model of care described in the articles adhered or did not adhere to each of the particular model components (codes). The authors met weekly to discuss the process of adherence. This discussion process was informed by adherence analyses [57]. During this process, authors were encouraged to identify any components that were potentially originally overlooked. No additional suggestions were made on key model components. The model components adhered to across the articles and models of care formed the basis of the results.

After a comprehensive list of the identified model components had been determined, two authors (KMK and AG) went through each article and identified them as either adhering or not adhering to each particular characteristic component, as determined by written evidence within the articles. This was done by having the two authors each providing their vote (i.e., adhering or not) and then comparing the two scoring. Any uncertainty in adherence assignment or discrepancies in voting was resolved through discussion amongst all the investigators as done in other reviews with similar methodologies [42].

### Step 6: Consultation

To further contribute to our component adherence, we shared our model components with the senior investigators of our peer-reviewed articles for feedback. We also asked the investigators to assess their level of agreement with our interpretations of their study's component adherence. Lastly, we asked authors to send along any studies that they believed would be relevant to our review. This was done via email by the first (KMK) and senior author (LS) in December 2022. After two months, we only received five replies from 13 potential authors (n = 5/13, 38%) and all five authors agreed with the adherence we provided their article with, suggesting an accurate adherence analysis. No investigators provided us with additional materials or feedback on the model components, rather just commenting on their article specifically.

## Results

**Fig. 1:**
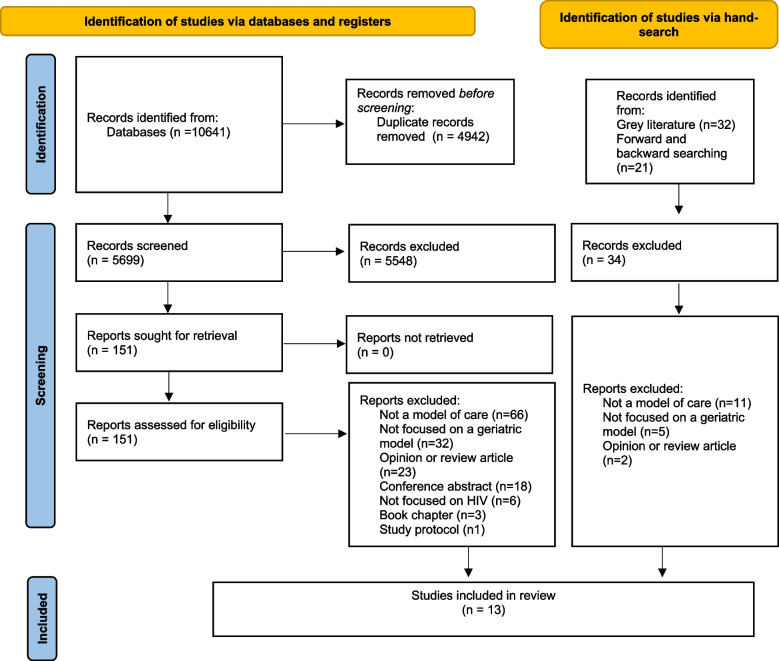
PRISMA flow chat diagram

The databases search yielded a total of 5699 unique citations, from which 151 articles were selected for full text review. Of these 151 articles, 12 peer-reviewed articles were included. An additional peer-reviewed article was obtained from hand searching. No grey literature was included. Thirteen articles were included in the final analysis (see Fig. 1 PRISMA flow chart).

Most (*n* = 10/13, 77%) of the publication activity occurred in the United States (USA) [28, 32–65]. The remaining three articles (*n* = 3/13,23%) were from the United Kingdom (UK)[66–68]. Over half (*n* = 9/13,69%) of the articles were published in the last 5 years (2018–2023) [28, 32–62]. In published papers, the most common research methods were qualitative. The key description from these studies were abstracted and are summarized in Table 1.

**Table 1:** Study details, organized alphabetically by author name

Author (year), Country	Objective(s)	Sample Size, Time Period	Patient Sex (n)	Patient Ethnicity, n (%)	Patient Age, Median or Mean, Range in Years	Healthcare Professional Roles Involved in Care	Intervention (Program Name)
Bitas (2019), [39] USA	To describe the recommendation patterns that arose from the CGA in a population of HIV-infected older adults, and to assess the adherence to recommendation during a 6-month follow-up	76 people, June 2013-July 2017	52 Male, 24 Female	40.8% African American 34.2% White 22.4% Latino	50 + Median age 67.2	HIV specialist (infectious diseases or internal medicine physician) Social worker, Psychiatrist Dietician Geriatrician	Comprehensive geriatric assessment (CGA) for older people at a HIV clinic
Cresswell [66] (2017), UK	To describe how widespread HIV services are in the UK and how they are organized	102 HIV clinics, Time Period NR	N/A	N/A	N/A	Physician HIV clinical nurse specialist Clinical psychologist Dietician Social worker Physiotherapist Occupational therapist	Electronic survey of HIV clinics using SurveyMonkey
Davis (2022), [41] USA & UK	To describe strengths and weaknesses of various models of geriatric consultation for older adults living with HIV	NR	NR	NR	50 +	Model 1: Geriatrician; HIV provider Model 2: Physician; Geriatrician with HIV training Model 3: Dual-trained provider with expertise in geriatrics & HIV	Model 1: Outpatient consultation Model 2: Combined HIV/geriatric multidisciplinary clinic Model 3: Dual-trained provider consultation
Garvey (1994), [59] USA	To describe issues in an existing model of care for elderly people with AIDS and suggest improvements	NR	NR	NR	50 +	Registered nurse (case manager) Social worker Counselor Therapist Nutritionist Home care aide Medical director Chaplain, volunteer(s)	Community-based delivery of hospice and home care with an on-call system. (**AIDS home care and hospice model of Visiting Nurses and Hospice of San Francisco**)
Greene (2018), [61] USA	To describe qualitative data from patients and providers that informed the development of a comprehensive care model for older people living with HIV	77 patients and 26 providers, March–April 2016	53 Male, 19 Female, 5 Transgender	50.6% Black 26% Non-Hispanic White 11.7% Latino 7.8% Other	50 + Median age 58 Range 50–77	Administration Nurse Medical assistant Nurse practitioner Physician	Focus groups and surveys to identify the most important health issues and/or needs facing older adults with HIV. (**Golden Compass**)
Greene (2020), USA	To evaluate the initial implementation of the Golden Compass program at San Francisco General Hospital	198 adults, January 2017-June 2018	178 Male, 20 Female	39% White 22% Black 7% Asian 5% American Indian/Alaska Native 17% Hispanic	50 + Mean age 62	Physician Medical director, Cardiologist Geriatrician Registered nurse Pharmacist Program coordinator Medical assistant	Implementation of a geriatric-HIV program using the RE-AIM framework (**Golden Compass**)
Heckman (2010), [60] USA	To test if a coping improvement group intervention could reduce depressive symptoms in persons over 50 years of age living with HIV/AIDS	295 patients, 12 × weekly sessions with 4 and 8 month follow up	197 Male, 96 Female	48% African American	50 + Mean age 55.3	NR	12 face-to-face group sessions on coping improvement
Heckman (2017), [62] USA	To determine trajectories of symptom change from two group tele-therapies for older people living with HIV and depression	105 patients, 12 week treatment period	Coping enhancement group: 28 Male, 26 Female Supportive-expressive group: 22 Male, 27 Female	Coping enhancement group: 27.3% White, 72.7% Persons of color Supportive-expressive group: 10.2% White, 89.8% Persons of color	Coping enhancement group: Mean age of 57.86 Supportive-expressive group: Mean age of 58.97	Therapist	12 weekly sessions of tele-therapy that were either 1) coping enhancement or 2) supportive-expressive
Levett (2020), UK	To describe the CGA approach used in a multidisciplinary HIV ageing clinic and the results of an initial evaluation of the clinic	52 patients, 2016–2019	47 Male, 5 Female	96% White	Mean age 67 Range 53–87	HIV physician Geriatrician HIV nurse specialist HIV pharmacist	Comprehensive Geriatric Assessment (CGA) used in an outpatient clinic (**Silver Clinic**)
Ruiz (2010), [64] USA	To describe the development of a geriatrics HIV screening program for patients over 60 years in an urban clinic; to report its initial results; and to discuss options for further development	17 referred to the program. Evaluations were done from May 2007 to May 2009. Target group selected in July 2009. Further screenings done yearly	9 Male, 8 Female Referred to program	NR	60 + Mean age 62.6	Dual-trained geriatrician/HIV specialist Social worker Pharmacist Nurse practitioner	Geriatric screening evaluations and subsequent referral to a geriatric HIV intervention program
Schmalzle (2022), [65] USA	To ​​describe the initiation and early outcomes of a new care geriatric model for older people with HIV	58 patients, March-June 2019	59% Male, 41% Female	95% African American	Mean age 59 Range 50–73	Social worker Pharmacists Physician	HIV primary and speciality care clinic in Baltimore, MD, USA (**THRIVE Program**)
Siegler (2018), [28] USA	To examine clinical programs for older people living with HIV internationally and compare their models	N/A	N/A	N/A	50 + N/A	Geriatrician Psychologist Pharmacist	N/A
Tan (2021), USA	To explore the impact of the Golden Compass program from the perspectives of both patients and primary care providers	13 patients, 11 primary care providers, October 2018-May 2019	Patients: 11 Male, 2 Female Providers: NR	Patients: 46.2% Black, 23.1% Other, 23.1% White, 15.4% Latnix, 7.7% Native Hawaiian/Pacific Islander Providers: NR	50 + \	HIV geriatrician Cardiologist Pharmacist General practitioner	Implementation of a geriatric-HIV program using the RE-AIM framework (**Golden Compass**)

### Patient population

Patients in the included models of care ranged from 48 [60]–87 years of age [67]. The number of patients served ranged from 76 [39] over 4 years to a maximum of 4000 at the time of data collection (period unspecified) [66]. Of those articles that reported sex (*n* = 9/13,69%), the majority described primarily male samples [39, 60–65, 68]. Articles that reported race/ethnicity (*n* = 7/13, 54%), described including participants who were mostly White [60, 61, 67] or African American [39, 62, 63, 65, 68]. These articles all included White individuals. Of the two (*n* = 2/13, 15%) studies that reported the median time since HIV diagnosis [39], the average was 12.5 [63]- 21.5 [39] years. Medicaid was used as the patients’ primary health insurance in the USA [39, 61, 62].

### Key operational components of geriatric models of HIV care

**Fig. 2:**
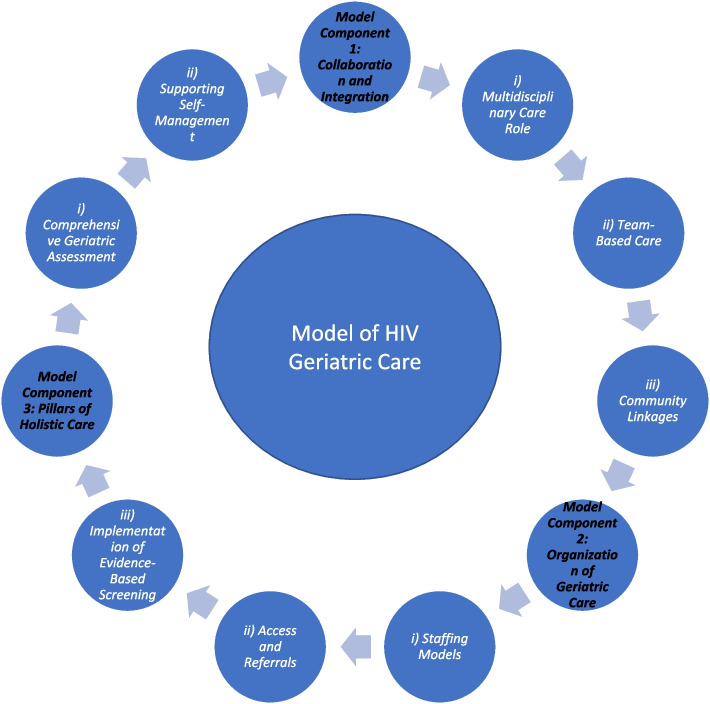
Main Model Components

The qualitative analysis identified three distinct model of care components, each with one or more sub-components. These components are listed and described in Table 2. Table 3 also lists the articles adherent to each component. These model components entail: Collaboration and Integration; Organization of Geriatric Care; and Support for Holistic Care. These three components are described and are illustrated in Fig. 2.

**Table 2:** Description of Model Components

Model Component	Description
*Model Component 1: Collaboration and Integration*	The organization and scheduling of planned care amongst various providers in health and community sectors to ensure effective intervention and care. Care is coordinated across healthcare and community settings
*i) Multidisciplinary Care Roles*	The involvement of healthcare providers from various disciplines in the delivery of care and the assignment of key roles among team members
*ii) Team-Based Care*	Providers working collaboratively as a team with defined tasks and responsibilities to provide effective care
*iii) Community Linkages*	How a model of care connects with community programs and services, and the partnerships formed with community organizations to deliver care and support
*Model Component 2: Organization of Geriatric Care*	The structures, procedures and policies of the healthcare system in which a geriatric model of care takes place
*i) Staffing Models*	The organization of healthcare professionals within a model of care
*ii) Access and Referrals*	How individuals living with HIV can access geriatric and specialized care, and the referral process to be seen by care providers
*iii) Implementation of Evidence-Based Screening*	The use of validated screening instruments to inform high-quality care
*Model Component 3: Support of Holistic Care*	How a model of care meets physical, spiritual, mental and social needs
*i) Comprehensive Geriatric Assessment*	All activities involved in the Comprehensive Geriatric Assessment of an older adult, that includes identifying medical, social and functional needs, and the development of an integrated care plan to meet those needs
*ii) Supporting Self-Management*	Any activities or strategies that help older adults living with HIV manage their own health concerns and be actively involved in their care

**Table 3:** Model Adherences, organized by study author name and grouped by program where appropriate

**Author (year)**	**1.Collaboration & Integration**	1.1 Multidisciplinary Care Roles	1.2 Team-Based Care	1.3 Comm-unity Linkages	**2.Organization of Geriatric Care**	2.1 Staffing Models	2.2 Access and referals	2.3 Implement-ation of Evidence-Based Screening	**3. Pillars of Holistic Care**	3.1 Comprehensive Geriatric Assessment	3.2 Supporting Self-Management
*Studies describing the AIDS home care and hospice model of Visiting Nurses and Hospice of San Francisco*											
Garvey (1994) [59	Yes	Yes	Yes	Yes	Yes	Yes	Yes	Yes	Yes	No	Yes
*Studies describing the Golden Compass Program*											
Greene (2018) [61]	Yes	Yes	Yes	Yes	Yes	Yes	Yes	Yes	Yes	Yes	Yes
Greene (2020) [60]	Yes	Yes	Yes	Yes	Yes	Yes	Yes	Yes	Yes	Yes	Yes
**Tan (2021)**	Yes	Yes	Yes	Yes	Yes	Yes	Yes	Yes	Yes	Yes	Yes
*Studies describing the Silver Clinic*											
Levett (2020) [67]	Yes	Yes	Yes	Yes	Yes	Yes	Yes	Yes	Yes	No	No
*Studies describing the THRIVE Program*											
**Schmalze (2022)**	Yes	Yes	Yes	Yes	Yes	Yes	Yes	Yes	Yes	Yes	Yes
*Studies describing othes singular models of care*											
Bitas (2019) [39]	Yes	Yes	Yes	Yes	Yes	Yes	Yes	Yes	Yes	Yes	Yes
Heckman (2010) [63]	No	No	No	No	Yes	Yes	Yes	Yes	Yes	No	Yes
Heckman (2017) [62]	No	No	No	No	Yes	Yes	Yes	Yes	Yes	No	Yes
**Ruiz (2010)**	Yes	Yes	Yes	No	Yes	Yes	Yes	Yes	Yes	No	No
*Studies describing multiple different models of care*											
**Creswell (2017)**	Yes	Yes	No	No	Yes	Yes	Yes	Yes	Yes	Yes	No
Davis (2022) [41]	Yes	Yes	Yes	Yes	Yes	Yes	Yes	Yes	Yes	Yes	No
Siegler (2018) [69]	Yes	Yes	Yes	Yes	Yes	Yes	Yes	Yes	Yes	Yes	Yes

### Model Component 1: Collaboration and integration

Eleven (*n* = 11/13, 85%) [28, 39, 41, 59–61, 64–68] articles described the importance of collaboration and integration for providers caring for older adults with HIV. Models of care frequently incorporated a team of multidisciplinary professionals from the health and social care sectors that were linked in with community supports to improve healthcare delivery for older adults with HIV.

### i) Multidisciplinary care roles

Multidisciplinary teams supported the care of older adults living with HIV in all eleven articles that adhered to the *Collaboration and Integration* model component (*n* = 11/13, 85%). These articles described several provider roles, including designated HIV specialists (infectious diseases or internal medicine physicians) [39, 41, 60, 61, 65–68], geriatricians [39, 41, 60, 61, 64, 65, 67, 68] and/or dual-trained HIV and geriatric physicians. Other physician roles included psychiatrists [39], endocrinologists [65], cardiologists [41, 60, 61, 68] and medicine fellows [64]. Numerous nursing roles [41, 59–61, 64, 65] were involved, such as HIV clinical nurse specialists [41, 66, 67] and nurse practioners [41, 64, 65]. Allied health professionals included dieticians [39, 65, 66]/ nutritionists[41], social workers[39, 41, 59, 61, 65, 66, 68], phsysiotherapists [41, 59, 66], occupational therapists [41, 59, 66], speech-language pathologists[59], counselors/therapists [59], homecare aides [59], clinical psychologists [65, 66] and specialist pharmacists [41–67].

In addition to healthcare providers, several models of care also included research team members (i.e. research coordinators [39], research assistants [39], graduate students in gerontology and epidemiology [41]), medical directors and administrative staff [59, 61] (e.g., program coordinator[60], a gerontologist [i.e., non-clinician] [41]), chaplains [59] and volunteers [59]. Peer navigator roles were also described [28, 41, 65, 68].

The key responsibilities of these providers differed between models of care and many had overlapping functions. Physicians [39, 41, 60, 61, 64–68] and nurses [41, 59–61, 64, 65] were often responsible for overseeing and ensuring appropriate medical care, such as disease and symptom management. Other healthcare professional roles and designated navigation-specific roles [28, 65, 68], provided medication, rehabilitation [41, 59, 66], dietary [39, 59, 65, 66], or emotional counseling to patients and caregivers [59]. Geriatricians, in particular, provided evidence-based, best-practice advice that was shared with patients’ primary care providers [39, 41, 60, 61, 64, 65, 67, 68]. HIV specialists generally oversaw HIV-related treatments and community services [39, 41, 60, 61, 65–68]. Pharmacists often provided medication instructions and explained care protocols [41, 60, 65–67]. All care providers were described as providing informational and tangible (i.e., hands-on care) support. Administrative and research staff were responsible for documenting relevant information accurately [39, 41, 59, 61]. Only one article mentioned the role of non-professional caregivers (i.e., spouse, partner, or friend) as part of the care team [59], in which they were described as providing much of the personal care involved in the home management of HIV [59].

Administrative team members and researchers support the collection of client information to systematically standardize clinical and research operations [39, 41, 59–61].

### ii) Team-Based care

Ten articles (*n* = 10/13, 77%) described the team-based delivery of multidisciplinary care, which was facilitated by several different mechanisms. Informational continuity was identified as being vital in ensuring a consistent and coherent approach to the management of older adults’ evolving needs [67]. A shared electronic health record was found to enable team-based care, including the ability for multiple providers to chat in real-time [28, 41, 60, 61, 68]. Moreover, the multidisciplinary team would often meet to discuss each patient’s background, their outcome measures, current clinical problems, and anticipated needs [28]. Consequently, the team would facilitate the appropriate screenings through access to different providers, services, and resources [28, 39, 41, 60, 61, 65, 68]. Following a referral and initial clinical visit, the HIV-geriatric specialists would maintain communication with the primary care team [28], make recommendations based on the identified age-related needs for care [28], initiate referrals to other specialist care providers and communicate with community stakeholders to meet other needs [59]. Team-based care allowed for all members of the circle of care to have a comprehensive knowledge of patients’ health and social care needs (e.g., functional, cognitive) [28]. Results from retrospective medical and pharmacy chart reviews helped inform all team decisions [65]. When deemed necessary, the team would be able to create a new action plan [39] and determine follow-up [64]. Nurses who worked in case manager roles helped to facilitate this care by coordinating a comprehensive, holistic care plan in collaboration with the patient, caregiver(s), physician(s), and other members of the care team [59]. Team-based models of care were felt to improve the coordination of care [41].

### iii) Community linkages

Nine articles (*n* = 9/13, 69%) described how the management of HIV in older adults involved active, collaborative partnerships between multidisciplinary healthcare providers and the various community resources available to individuals living with HIV. Models of care were often delivered in linkage with community resources (e.g., social groups) [41] and through community partners (e.g., volunteer organizations) [41]. Social workers often helped to facilitate community linkages [59], and grant-funding helped to pay for community services [65]. By working with community partners [41], models of care were able to deliver both nonclinical care [39] (e.g., peer support to decrease isolation and depression [41]), as well as clinical care [28] (e.g., care facilitated by a community nurse [39]). Community outreach also helped to foster friendships amongst older adults living with HIV through social and community-building activities including dinners, speeches, dances, and trips [59]. Local partner agencies assisted with meeting the housing needs for patients with marginal housing [61], and with the provision of legal services [61]. Partnering medical HIV-geriatric services with community services was thought to result in improved access to services [28], reduced social isolation [60], improved home safety management [59] and the provision of spiritual care such as priests, rabbis, or pastoral personnel [59].

### Model Component 2: Organization of geriatric care

The specific organizational structure of each model of care varied, particularly as it related to staffing models, processes for access and referrals, and the implementation of evidence-based, best-practice care and follow-up. All articles adhered and contributed to this model component. Models of care were often delivered through clinics that were predominantly hospital-based (i.e., operating within a hospital) [39, 60, 61, 65–67]. Additionally, geriatric clinics were outpatient clinics housed within existing HIV clinics [41] or community-based services providing home care [59]. Some models of care were able to be delivered virtually, either solely via phone [62] or in addition to in-person delivery [65, 66]. Some clinics ran weekly [66], bi-weekly [65] or monthly [41–67], whereas others were full-time [39, 65].

### i) Staffing models

Within the identified models of care, various staffing models were described. All articles contributed to this sub-component. The Geriatrician-Referral model included a geriatrician who consulted on patients [39, 41, 60, 61, 64, 65] based on a referral from the primary care team (often an HIV provider [41]), according to the perceived need (e.g., cognitive concerns). Six articles (*n* = 6/13, 46%) adhered to this. The Joint-Clinic model involved a geriatrician and HIV physician who were present in a single, combined clinic [41, 66–68]. Four articles (*n* = 4/13, 31%) adhered to this model. The HIV-Physician-led model involved staffing clinics with a HIV physician and clinical nurse specialist trained in geriatrics, without geriatrician involvement [65, 66]. Two articles (*n* = 2/13, 15%) adhered to this model. A further staffing model, the Dual-Trained Provider model, involved a dually-trained HIV and geriatrics provider, as either a physician [41, 68] or psychotherapist [62, 63]. Four articles (*n* = 4/13, 31%) adhered to this model. The Nurse-led model, involved nurse-lead teams of allied health professionals [59]. Only one article (*n* = 1/13, 8%) adhered to this model [59].

### i) Access and referrals

All articles described processes to ensure appropriate access to care, and thus contributed to this sub-component. Referrals and on-call services [59] were used to facilitate access to care [59]. In some models of care, older adults were only able to access geriatric services via a referral from their HIV primary care team [39, 41, 60, 61, 67], while in other models, referrals were triggered by a combination of age (i.e., 50 years of age or older) and need (e.g., complexity) [28, 66–68]. The process of receiving geriatric care often began with an assessment of patients’ needs and functional status (e.g., cognition) [39] and the collection of demographic information (e.g., age, sex, race/ethnicity, HIV risk factors, marital status, insurance status [39])[28, 61, 65]. Provider referrals were often documented through tracking scheduled appointments [60, 61, 68], however, limitations of this method included HIV providers not remembering to refer [41] and patient barriers such as confusion over the need for the referral which may result in skipping geriatric appointments [41]. One model of care implemented patient reminders to help ensure appointments were attended [64]. Two articles (*n* = 2/13, 15%) relied on referrals through an AIDS service organization [62, 63]Moreover, across the models, patients could choose to be referred to one service (e.g. cardiology clinic) or multiple (e.g., geriatrics clinic) [60, 68]. Patients could choose to have follow up with the geriatrician[28] and/or be connected with a primary care provider [41]. Clinics have developed guidelines and policies to guide the operation of services [28].

### ii) Implementation of evidence-based screening

All articles described the incorporation of gold-standard, evidence-based screening practices into their geriatric care. Mood symptoms were assessed using the Hospital Anxiety and Depression Scale [60, 62, 63, 67], the Geriatric Depression Scale [62, 63], the Older Peoples’ Quality of Life Questionnaire [67] and/or the Patient Health Questionnaire [39], while cognition was assessed using tools such as the Montreal Cognitive Assessment [60]. CGAs were followed up with direct actions such as counseling (e.g., about ageing) [28, 39, 60], assessments of comorbidities, age-appropriate preventative health screening[41, 60, 61], and pharmacist reviews targeting polypharmacy and drug safety [4, NaN]. In addition to the CGA, clinics offered British HIV Association (BHIVA)-recommended screening (i.e., guidelines for the management of HIV), an antiretroviral review, a functional review and full medication review [28, 66]. Emotional support was monitored using the ‘Therapy Content Checklist’ [62, 63]. The goal of using valid measurements was to promote best practice [59].

### Model Component 3: Support for holistic care

As older persons are more likely to experience cumulative health challenges that affect their quality of life, models of care for people ageing with HIV have incorporated a comprehensive holistic management approach. All included articles adhered and contributed to this model component. Clinics provided care for patients with multimorbidity [60, 61, 66, 67] and helped them to overcome socioeconomic challenges [41], substance use disorders [60, 65] and social isolation [60, 62, 63] by understanding their backgrounds[41]. Physical health consultations considered cardiovascular disease, dental health, eye health and bone health[28, 41, 60, 61, 64, 68] to address HIV and metabolic-related complications [41]. Care plans incorporated medication prescriptions [28, 39, 60, 61, 66–68], preventative screening [28, 39, 60, 61, 64–68], age-related disease processes (e.g., cognitive-testing) [28, 39, 41, 59–61, 64–68], psychosocial interventions to improve social networks and mental health [28, 39, 59, 60, 62–65], exercise and nutrition regimens [39] and behavioural health supports (e.g., smoking cessation, therapy) [28, 39, 59–64, 67] to meet the holistic needs of each patient. Spiritual support delivered through religious leaders, mental health counselors/therapists, and emotional support volunteers was also offered [59, 64].

### i)Comprehensive geriatric assessment

Most models of care (*n* = 8/13,61.5%) involved a CGA [28, 39, 41, 60, 61, 66, 68] or utilized geriatric screening tools [65] to guide holistic care plans. Most CGAs were delivered by geriatricians who would write full consultation notes [39, 60, 61], although non-geriatrician health care providers were often trained to administer geriatric screening tests [41, 64]. The CGA provided an overview of physical and mental health, as well as social support systems [39], using validated scales [39].

### ii)Supporting self-management

The models of care in six articles (*n* = 6/13, 46%) aimed to support the self-management of older adults living with HIV. The goal of self-management was to enable patients to better manage their health outside of the clinic setting by involving older adults in medical decision-making [60, 68] and managing their chronic illnesses [59–61]. Self-management involved education [39, 59, 60, 65] and coaching [28] about health behaviours, guidance for choosing appropriate interventions [39, 59, 65] to improve a patient’s health status [28, 65], and increased health care utilization to improve patient involvement in care [60, 65]. Some models involved classes where older adults could learn about various health conditions [60–63]. Where self-management was not possible due to cognitive or functional impairments, healthcare professionals provided education to individuals’ social support networks such as to encourage their inclusion in care [39, 59]. To evaluate self-management, some studies included surveys about knowledge in the evaluations of the clinic models [60, 61].

## Discussion

Our scoping review of the literature identified thirteen articles describing geriatric models of care for older adults living with HIV. The identified models came from two countries, the USA and the United Kingdom, and incorporated screening for geriatric syndromes [28, 39, 41, 60, 61, 65, 66, 68]. From these articles, we identified three overarching key model components: Collaboration and Integration; Organization of Geriatric Care; and Support for Holistic Care. The models of care were largely delivered by a consulting geriatrician [39, 41, 60, 61, 64, 65] via a referral from an HIV provider [41], from a joint clinic model involving a geriatrician and HIV physician[41–68], or through a dually-trained HIV-geriatrics provider [41, 62, 63, 68]. However, some models did not involve a geriatrician [59, NaN]. Table 4 summarizes the future recommendations from the included articles.

**Table 4:** Recommendations for Future Research

Recommendations for Future Research Derived from Included Studies
• Include a control group (Bitas et al., 2019) • Include participants of diverse ages in evaluations (e.g., ‘the oldest-old’) (Bitas et al., 2019), to help determine who would benefit from services the most (Greene et al., 2020), include comprehensive geriatric assessments (Bitas et al., 2019; Levett et al., 2020) • Use Delphi approaches to gain consensus on how issues of ageing should best be addressed in the context of HIV (Cresswell et al., 2017) • Identify strategies for ensuring funding within the context of model spread and sustainability, including differences that exist in single-payer systems compared to multi-payer systems (Davis et al., 2022; Schmalzle et al., 2022) • Include more patient-reported outcomes, including self-reports of health (Greene et al., 2018; 2020) and the perspectives of surrogate decision-makers (Schmalzle et al., 2022) • Explore implementation differences in urban and rural settings (Greene et al., 2020) • Conduct analyses of symptom relief, such as, depressive symptom relief over longer-term follow-up (e.g., 4-month and 8-month follow-up) (Heckman et al., 2010; 2017) • Include outcomes related to mobidity and mortality in studies (Ruiz et al., 2010)
• Explore opportunities to foster collaboration with local governments, insurers, and foundations to co-develop and test novel programs (Seigler et al., 2018)

Our review identified that most models of geriatric-HIV care are delivered by multidisciplinary teams that facilitate integrated health and social care. Multidisciplinary providers who work in team-based care models have been shown to improve clinical outcomes among HIV patients [70–73]. This study provided examples of collaborations in which practitioners worked together to meet the diverse needs of patients. Our data expand this finding by suggesting that multidisciplinary care providers help to facilitate referrals to even more providers, particularly those working in community settings, to ensure care continuity and care coordination to meet holistic needs for support. However, it is important for future research to further understand what staffing model of multidisciplinary team care contributes best to the quadruple aim of optimizing health system performance (i.e., improving the individual experience of care; improving the health of populations; reducing the per capita cost of healthcare and creating better provider experiences [74]) and the limitations of the existing approaches. Moreover, given the shortage of geriatricians [45] to meet patient needs, it is important to consider the transferability of models that involve a geriatrician [39, 41, 60, 61, 64, 65][66–68], or dually-trained HIV-geriatrics provider [41, 62, 63, 68].

The increasing proportion of older adults living with multimorbidity, including HIV, has evoked calls for tailored geriatric services that respond to their evolving needs. Our results suggest that care delivery should address multiple complex and multidimensional aspects of health and wellness, including psychosocial needs such as strategies to reduce social isolation. However, none of the articles discussed the provision of palliative or hospice care. Palliative care has been posited to augment HIV patients’ health and social care outcomes [75]. Implementation science may help researchers identify how to implement novel palliative care interventions into exiting practices and support uptake and sustainability by considering why, how and in what circumstances barriers and facilitators may be present [76]. In addition, older adults were described as being decision makers in their care such as being able to choose the follow up services they receive [60, 68]. While some programs sought the input of older adults (e.g., through focus groups, none explicitly mentioned partnering with older adults to co-design their models of HIV care. Other HIV interventions have included individuals living with HIV on their steering committees and in development teams, such that care meaningfully reflects their wishes and preferences [77–79]. These interventions do not include older adults. Future models of care may wish to engage older adults in co-design to conceptualize and brainstorm program delivery [80, 81].

Our review identified several areas of research with limited information. Most literature was published in the USA. Only one article mentioned the role of family caregivers in the care of HIV [59]. However, individuals living with HIV may receive support from non-kin family caregivers, such as friends [82]. Research is needed to better understand how broader conceptualizations of family can be embedded into the multidisciplinary care teams to help facilitate family-centered care [43, 83]. Moreover, none of the articles mentioned care being delivered in the context of nursing or long-term care homes, nor did they mention offered referrals to long-term care facilities or services. Research is needed to determine the optimal approach for delivering geriatric services in long-term care settings to older adults living with HIV. Strategies are also needed to effectively embed HIV care into the already overburdened and under-resourced long-term care sector. While telehealth has proven to be an effective strategy for delivering HIV care [84, 85], particularly in rural and remote communities where specialists may not be readily available [86], additional research is needed to identify the best practices and limitations for delivering geriatric-focused models of care virtually. Lastly, no studies have evaluated how to best incorporate culturally-sensitive geriatric care across racial and ethnic groups [87, 88]. Thus, more data are needed to develop culturally-informed models of care to better engage and care for diverse populations of older adults living with HIV, particularly for adults with certain racial and ethnic backgrounds who may face pervasive stigma for accessing HIV care [89, 90].

### Limitations

As with any review, our findings must be considered within the context of the limitations. Despite our best efforts (i.e., multiple databases, peer-reviewed strategy, screening in duplicate, bibliographic searches, contacting authors of the reviewed articles), we may have inadvertently missed potentially relevant articles. Moreover, we may have missed papers of programs not yet described in the literature, such as those recently funded or piloted. Similarly, we limited the inclusion criteria to studies available in English due to resource constraints (i.e., lack of funding to support translation) and, consequently, may have biased our included studies to those published in English-speaking countries [91]. However, the intention of scoping reviews is to provide an overview or “map” of the breadth of existing literature, and thus, future exploration is warranted that builds upon our search strategy. Studies focused on individuals with HIV, but did not include description of older adults living with co-morbidities that impair healthcare decision-making, such as dementia, making it difficult to comment about models of care for individuals who require decision-making support. Lastly, stakeholders in implementing, delivering and receiving models of care (e.g., individuals with HIV, policy-makers, healthcare professionals) were not involved in the study design nor analysis.

## Conclusions

Our review suggests that novel models of geriatric care for older adults living with HIV should include collaboration and integration, an organization of care that considers appropriate and timely referrals, communication of medical information and the implementation of evidence-based recommendations, as well as a holistic understanding of the dimensions of care, such that they support self-management. This proposed geriatric-based model can provide the framework to inform future implementation science and evaluative research to support further refining and developing this model. However, further research is needed to inform models of geriatric-HIV care in long-term care settings. Given the increasing number of older adults living with HIV, the development of best-practice models of integrated care can hopefully guide healthcare professionals to provide optimal care in the context of the complexities of care for older adults with HIV.

## Supplementary Information

**Additional file 1.**

## Data Availability

The analysis files and data used and/or analyzed during the current study are available from the corresponding author on reasonable request.

## Abbreviations

CGA: Comprehensive Geriatric Assessment
HIV: Human Immunodeficiency Virus
